# SIRT1–Redox Crosstalk in Aging: Adaptive Capacity and Cellular State Transitions

**DOI:** 10.3390/biology15201845

**Published:** 2026-10-14

**Authors:** Mengting Yan, Linrui Hua, Yun He, Yanzhou Li, Qun Yang, Yuncai Chen

**Affiliations:** 1 Department of Anatomy, School of Medicine, Yangtze University, Jingzhou 434023, China; yanmengting.stu@yangtzeu.edu.cn (M.Y.);; 2 Department of Medical Imaging, School of Medicine, Yangtze University, Jingzhou 434023, China

**Keywords:** SIRT1, redox homeostasis, adaptive homeostasis, NAD^+^ metabolism, mitochondrial function, cellular stress response, cellular senescence, aging

## Abstract

Aging gradually reduces the ability of cells to maintain and restore homeostasis under metabolic and oxidative stress. Sirtuin 1 (SIRT1) is an NAD^+^-dependent deacetylase that regulates metabolism, mitochondrial function, inflammatory signaling, and cellular stress responses. Its activity depends on NAD^+^ availability, redox state, post-translational regulation, subcellular localization, and metabolic context, arguing against a simple linear relationship between oxidative stress, SIRT1 loss, and cellular dysfunction. SIRT1 functional reserve is defined as the remaining capacity of SIRT1-dependent processes to respond to a defined cellular demand and is placed within a broader framework of adaptive capacity and adaptive threshold. An experimental protocol with measurable readouts is described to test this framework. SIRT1–redox–mitochondrial adaptive capacity is examined under transient, repeated, and persistent stress, with attention to incomplete recovery and transitions from adaptive to compensatory and persistent dysfunctional states. Neuronal vulnerability is also considered in relation to mitochondrial dependence and proteostasis. Rather than treating SIRT1 activation as universally beneficial, strategies that restore the conditions required for appropriate SIRT1 activity are discussed. The proposed framework remains a hypothesis and requires experimental and clinical validation.

## Simple Summary

Aging gradually reduces the ability of cells to respond to stress and return to a stable state. Sirtuin 1 (SIRT1) links cellular metabolism with stress responses, inflammation, mitochondrial function, and cell survival. Its effects are not always protective and depend on the cellular environment and the type and duration of stress. This review examines how SIRT1 interacts with redox and mitochondrial processes during aging, with a focus on NAD^+^ availability, mitochondrial function, redox balance, protein regulation, and recovery after stress. An adaptive-capacity framework is proposed in which cells may move from effective adaptation to compensation and, when recovery remains incomplete, to persistent dysfunction. Within this framework, SIRT1 functional reserve is introduced as a measurable concept describing the remaining capacity of SIRT1-dependent processes to respond to cellular stress. This perspective may help explain why increasing SIRT1 activity does not always improve aging-related outcomes. Future interventions may therefore need to restore the cellular conditions that allow SIRT1-dependent responses to function appropriately.

## 1. Introduction

Aging gradually reduces the ability of cells and tissues to maintain and restore homeostasis under metabolic and environmental challenges. Redox regulation is one of the systems affected by aging. Reactive oxygen species (ROS) can act as signaling molecules under physiological conditions but may damage cellular components when their production, removal, or compartmentalization becomes dysregulated [1,2,3,4]. The effects of redox imbalance depend not only on ROS abundance but also on stress intensity and duration, cellular metabolic state, and the ability of the cell to recover.

Sirtuin 1 (SIRT1) is an NAD^+^-dependent deacetylase that links cellular metabolism with stress responses, mitochondrial function, inflammatory signaling, and cellular senescence [5,6]. Its activity depends on NAD^+^ availability [7], but SIRT1 protein abundance alone does not fully reflect its functional state. Post-translational modifications, protein interactions, substrate access, subcellular localization, and protein stability can all influence SIRT1-dependent signaling [7,8,9,10]. Previous reviews have often described SIRT1 within a linear antioxidant model in which oxidative stress is placed upstream of reduced SIRT1 activity, followed by impaired cellular protection and dysfunction [6,11,12,13,14]. This model provides a useful starting point but does not fully explain why SIRT1 manipulation can produce different outcomes across cell types, tissues, and stress conditions. The biological effects of SIRT1 during aging may therefore depend not only on its abundance or activity but also on whether the cell can engage, coordinate, and recover SIRT1-dependent responses as metabolic and redox demands change.

**Figure 1:**
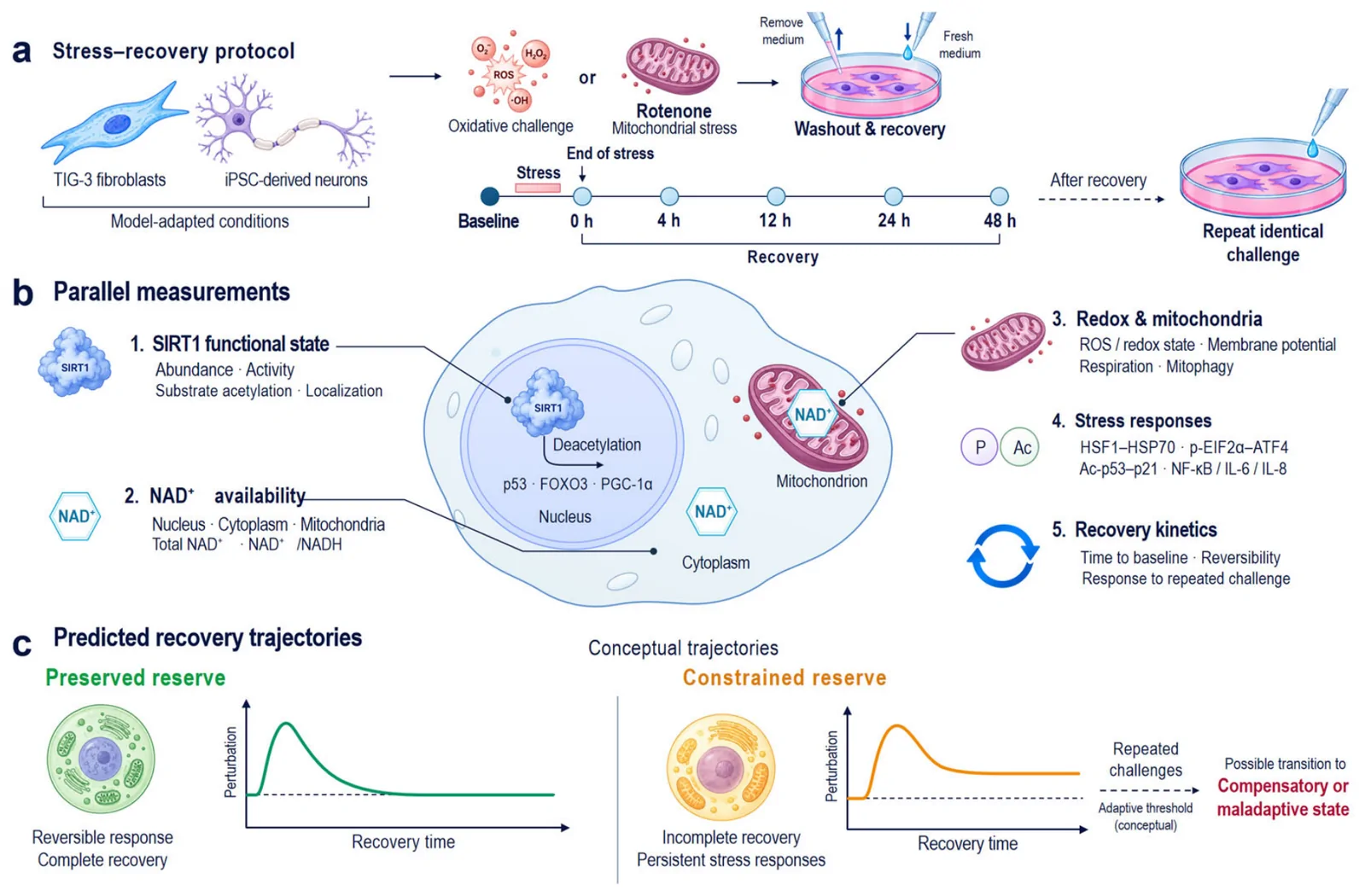
Schematic experimental protocol for operationalizing SIRT1 functional reserve and adaptive threshold. (**a**) Stress–recovery protocol. (**b**) Parallel measurements. (**c**) Predicted recovery trajectories.

Three concepts organize the framework used in this review. SIRT1 functional reserve refers to the remaining capacity of SIRT1-dependent regulation to respond to a defined cellular demand. It is a conceptual construct that can be operationalized through measurable parameters, including NAD^+^ availability, catalytic activity, substrate-specific deacetylation, post-translational regulation, subcellular localization, protein stability, and recovery kinetics. Adaptive capacity refers to the ability of a cell to mount, coordinate, and recover from stress responses while maintaining or restoring homeostasis. In contrast, adaptive homeostasis describes the transient expansion or contraction of the homeostatic range in response to changing conditions [15,16]. Adaptive capacity therefore encompasses not only adjustment of the homeostatic range but also the ability to sustain an appropriate response and recover after stress. Unlike adaptive capacity, which represents a broader cellular-level ability to maintain and restore homeostasis, SIRT1 functional reserve is proposed to describe the remaining operational flexibility of SIRT1-dependent regulatory processes under a defined stress demand. Biological resilience generally refers to the ability of a biological system to maintain or recover function following perturbation, whereas SIRT1 activity represents a measurable enzymatic state at a given time point. Therefore, SIRT1 functional reserve is proposed as an intermediate molecular-level concept that links SIRT1-dependent regulation with broader adaptive capacity and resilience. The adaptive threshold is treated as a conceptual boundary at which stress burden begins to exceed the coordinated capacity of these systems. It is not a fixed molecular switch. Depending on stress intensity, duration, repetition, and cellular context, transient stress may be resolved, whereas repeated or persistent stress may increase compensatory demands and, when recovery remains incomplete, contribute to persistent dysfunction. The review focuses on SIRT1–redox–mitochondrial adaptive capacity rather than on all aspects of SIRT1 biology or all hallmarks of aging. The experimental protocol presented in Figure 1 and Table 1 provides a practical framework for testing these concepts.

This review adds three main elements to previous discussions. First, it defines SIRT1 functional reserve as a molecular property that can be studied through NAD^+^ availability, catalytic activity, substrate-specific deacetylation, subcellular localization, and recovery kinetics. Second, it places SIRT1–redox–mitochondrial interactions within an adaptive-capacity framework while considering tissue and stress context. Third, it translates the proposed model into testable predictions and a practical experimental protocol. Together, these elements provide a testable framework for understanding how SIRT1-dependent regulation influences cellular adaptation under changing metabolic and redox conditions.

## 2. Redox Stress as an Adaptive Challenge: Establishing the SIRT1 Adaptive Landscape

### 2.1. Redox Imbalance as an Adaptive Challenge

Aging alters cellular redox regulation, but this does not necessarily mean a continuous increase in oxidative damage. Redox homeostasis depends on oxidant production, antioxidant and repair systems, cellular metabolism, and processes that are specific to different cellular compartments. Oxidative stress is therefore better understood as a disturbance in the balance between reactive species and the systems that control their production, removal, and biological effects [26,27]. In this review, the term “redox” is used broadly to include reactive oxygen species (ROS) and reactive nitrogen species (RNS), as well as NAD^+^/NADH, NADP^+^/NADPH, GSH/GSSG, and thioredoxin systems. It also encompasses compartment-specific redox processes and redox-dependent modifications such as cysteine oxidation and S-nitrosylation. This definition is consistent with the concept of the “redox code” [27] and with current models of redox regulation [3].

ROS are generated during mitochondrial respiration and through enzymatic and non-enzymatic reactions in several cellular compartments [28,29]. Mitochondrial ROS are not necessarily damaging. At controlled levels, they can function as signaling molecules and participate in cellular responses to metabolic and environmental changes [30]. Their effects depend on the chemical species involved, the site of production, local antioxidant capacity, and the magnitude and duration of the stress [26,27,28]. Aging-related changes in mitochondrial function, metabolism, proteostasis, and inflammatory signaling can affect both reactive-species production and the ability of cells to buffer or resolve redox stress. Redox signals can therefore contribute to adaptive responses, whereas persistent or poorly resolved oxidative stress can impair macromolecular integrity and cellular function [26,30,31]. Antioxidant defenses are also dynamic and regulated systems, and their effectiveness depends on when and where reactive species are produced and on the capacity of the relevant redox systems to respond [32].

SIRT1 is one component of this response to metabolic and oxidative challenges. As an NAD^+^-dependent deacetylase, it connects cellular metabolic state with stress-responsive signaling. Its function depends not only on protein abundance but also on NAD^+^ availability, post-translational regulation, redox-dependent modification, and cellular context. SIRT1–redox interactions are therefore better considered within a broader adaptive landscape than as a simple sequence in which oxidative stress suppresses SIRT1 and subsequently causes cellular damage.

### 2.2. NAD^+^ Availability as a Constraint on SIRT1 Activity

SIRT1 activity requires NAD^+^, providing a direct link between cellular metabolism and SIRT1-dependent signaling [33,34]. NAD^+^ is continuously synthesized and consumed, and its abundance reflects the balance among biosynthesis, recycling, transport, and consumption [35,36]. Sirtuins, PARPs, and CD38 all consume NAD^+^, with CD38 being particularly relevant to aging and immune-cell remodeling [37]. Changes in NAD^+^ concentration therefore reflect the combined effects of several processes rather than a single upstream defect. DNA damage, for example, activates PARP enzymes and increases NAD^+^ consumption, while experimental studies have also linked NAD^+^ availability to nucleotide metabolism and genomic DNA replication [38]. Increased CD38 activity in aging-associated immune cells has been associated with changes in NAD^+^ and nicotinamide mononucleotide levels [37].

Total cellular NAD^+^ should not be regarded as a direct measure of SIRT1 activity. NAD^+^ metabolism is spatially organized, and different cellular compartments can maintain distinct NAD^+^ states. Recent work indicates that these pools are connected and that mitochondrial NAD^+^ can buffer cellular NAD^+^ availability when consumption increases [39]. Bulk measurements may therefore fail to capture the local NAD^+^ environment experienced by SIRT1. Age-related changes in NAD^+^ also differ among tissues and species and may depend on experimental conditions and measurement methods [34,40]. The statement that NAD^+^ invariably declines with age is consequently too broad. A more cautious interpretation is that aging alters NAD^+^ metabolism and availability in specific cellular contexts [40,41].

For SIRT1, NAD^+^ availability may act as an important constraint rather than a direct readout of enzymatic function. Even when NAD^+^ is available, SIRT1 activity can be affected by post-translational modification, protein interactions, substrate accessibility, and intracellular localization. Changes in NAD^+^ availability may alter SIRT1-dependent signaling without producing a proportional change in SIRT1 protein abundance. Protein abundance alone may therefore provide an incomplete picture of age-related changes in SIRT1 function. Alternative splicing adds another layer of regulation. The full-length SIRT1-v1 isoform supports oxidative phosphorylation and ATP production by upregulating mitochondrial respiratory complex I, whereas the v2 and v3 isoforms, which lack part of the N-terminal region, show reduced activity [22]. Because these shorter isoforms tend to accumulate with age, the functional composition of SIRT1 may change even when total SIRT1 protein remains relatively stable. SIRT1 abundance and SIRT1 functional reserve should therefore not be considered equivalent.

SIRT1 is also regulated by post-translational modifications, protein stability, protein–protein interactions, and redox-dependent changes. Oxidants and carbonyl stress can modify SIRT1 and reduce its enzymatic activity, with cysteine modification and altered protein stability implicated in this response [42]. Direct oxidative modification, however, represents only one layer of regulation. Redox stress can also affect SIRT1 indirectly through changes in NAD^+^ metabolism, DNA damage responses, mitochondrial function, and inflammatory signaling. These mechanisms may occur at the same time, making it difficult to attribute a change in SIRT1 activity to a single cause. Stress-responsive modifications may also preserve or enhance SIRT1 activity without increasing its expression. For example, O-GlcNAcylation of SIRT1 increases its deacetylase activity under stress and contributes to cytoprotection in experimental models [43]. The direction and magnitude of SIRT1 responses thus depend on the relationship among redox state, metabolic status, NAD^+^ availability, and regulatory modifications. Measuring SIRT1 abundance alone provides an incomplete picture of its functional state [42,43,44,45].

### 2.3. Beyond the Linear SIRT1–Antioxidant Model

A common model places oxidative stress upstream of reduced SIRT1 function, followed by impaired antioxidant, metabolic, inflammatory, and mitochondrial responses [6,11,12,13,14]. This model provides a useful starting point, but it does not fully explain why SIRT1 manipulation can produce different outcomes across tissues, cell types, and stress conditions. ROS can function as signaling molecules under physiological conditions, whereas excessive or persistent redox stress may contribute to molecular damage [30,46,47]. Similarly, SIRT1 activity depends on NAD^+^ availability, subcellular NAD^+^ distribution, post-translational regulation, and substrate accessibility rather than on protein abundance alone [39,44,45,48]. The consequences of SIRT1 activation therefore depend on the cellular environment and on whether stress responses can be effectively resolved.

Adaptive homeostasis describes transient changes in the homeostatic range in response to changing conditions [15,16]. Adaptive capacity is used here to describe the ability of a cell to mount, coordinate, and recover from stress responses while maintaining or restoring homeostasis [16,49,50,51]. Adaptive capacity extends this concept by considering the ability to sustain an appropriate response and recover after stress, including situations in which recovery remains incomplete. Within this broader process, SIRT1 functional reserve refers to the remaining capacity of SIRT1-dependent regulation to meet a defined cellular demand. It is not a single measurable value but can be examined through NAD^+^ availability, SIRT1 catalytic activity, substrate-specific deacetylation, subcellular localization, and recovery after a defined stress.

**Figure 2:**
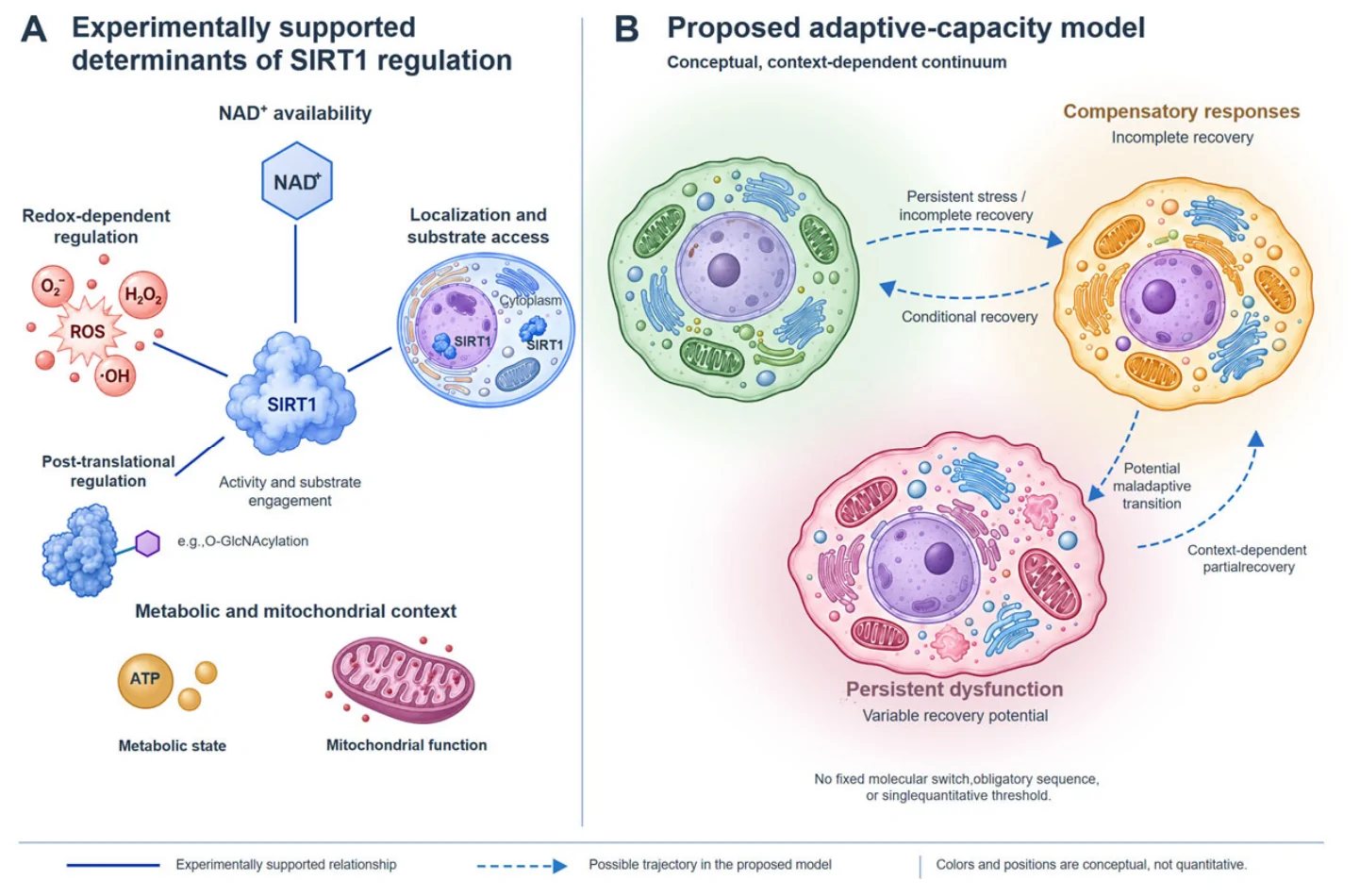
Proposed SIRT1-dependent adaptive-capacity model during aging. (**A**) Experimentally supported determinants of SIRT1 regulation, including NAD^+^ availability, redox-dependent regulation, subcellular localization and substrate access, post-translational regulation, and metabolic and mitochondrial context. (**B**) A conceptual, context-dependent continuum illustrating adaptive, compensatory, and persistent dysfunction states. The model is intended as a conceptual framework rather than a fixed sequence of molecular events and does not imply a single molecular switch or quantitative threshold. State transitions and recovery may vary with stress intensity, duration, repetition, cellular context, and the extent of recovery Transitions between states are not necessarily unidirectional, and persistent dysfunction should not be equated with irreversible commitment, as reversibility has been observed under specific experimental conditions.

SIRT1 functional reserve therefore differs from SIRT1 activity, which describes enzyme output at a given time, and from adaptive homeostasis or biological resilience, which describe broader cellular responses [50,51,52,53]. This distinction allows SIRT1-dependent regulation to be examined in relation to cellular stress adaptation and recovery. The usefulness of this concept will depend on whether these measurements can distinguish preserved SIRT1 function from reduced functional flexibility across cellular contexts. The adaptive landscape proposed in this review does not represent a fixed sequence of molecular states. Instead, cellular responses may remain reversible, require increasing compensation, or progress toward incomplete recovery and persistent dysfunction depending on stress intensity, duration, repetition, and cellular context. Figure 2 illustrates these possible states and transitions.

## 3. Adaptive-to-Compensatory Transition: Remodeling of Cellular Stress Responses

### 3.1. Transient Stress and Protective Adaptation

Cells do not always respond to stress in direct proportion to the amount of damage. Modest stress can induce reversible changes in metabolism, gene expression, protein turnover, and antioxidant defenses that help cells adjust to altered conditions. Adaptive homeostasis describes this temporary adjustment in the homeostatic range [15,16,54,55]. The response is generally reversible, and its benefit also depends on reducing stress-protective activity when the stress has resolved [56,57,58]. Aging can reduce this flexibility and increase vulnerability to subsequent stress [16,59,60,61].

SIRT1 participates in several systems that support this adaptive response. SIRT1 regulates the heat shock response through deacetylation of heat shock factor 1 (HSF1), and SIRT1 activation can prolong HSF1 binding to heat shock-responsive promoters [62]. SIRT1 also interacts with energy-sensing pathways. AMPK and SIRT1 regulate PGC-1α during fasting and exercise, and AMPK signaling can influence SIRT1-dependent metabolic adaptation in skeletal muscle [63]. The AMPK–SIRT1 relationship is bidirectional: AMPK activation can increase NAD^+^ availability through effects on fatty acid oxidation and NAMPT, thereby supporting SIRT1 activity and deacetylation of PGC-1α, FOXO1, and FOXO3a. Conversely, SIRT1 can deacetylate LKB1 and promote AMPK activation [63]. This reciprocal interaction links cellular energy status to transcriptional adaptation. With aging, changes in AMPK signaling and NAD^+^ availability may alter this coordinated response. Importantly, changes in SIRT1 activity do not necessarily parallel changes in protein abundance. In human skeletal muscle, high-intensity interval exercise increased SIRT1 activity despite a reduction in total SIRT1 protein [64,65]. This finding further supports the distinction between SIRT1 expression and its functional state.

SIRT1 also contributes to mitochondrial adaptation. The effect can depend on the SIRT1 isoform: SIRT1-v1 increases complex I activity, whereas isoforms lacking part of the N-terminal domain show lower activity [22]. SIRT1 can also regulate mitochondrial biogenesis through PGC-1α deacetylation, and pharmacological SIRT1 activation has been associated with improved mitochondrial recovery after acute oxidant injury [66,67]. In a zebrafish model of muscle atrophy, *Sirt1* inhibition impaired the mitochondrial unfolded protein response and increased protein aggregation [68] (Table 2).

These findings indicate that SIRT1 can support adaptation through metabolic remodeling, proteostasis, mitochondrial maintenance, and stress-responsive transcription. They do not establish SIRT1 as the main determinant of adaptive capacity. Its effects depend on stress type and duration, cellular metabolism, and the surrounding regulatory network [69,70]. Increased SIRT1 activity does not necessarily indicate greater adaptive capacity, just as reduced activity does not necessarily indicate an immediate loss of resilience. A strong stress response indicates that a cell has detected a challenge but does not by itself demonstrate functional recovery. Conversely, a weaker response may reflect successful resolution rather than failed protection [71,72,73].

**Table 2:** Representative experimental evidence informing the SIRT1-dependent adaptive-capacity framework.

Regulatory Dimension	Experimental Model and Manipulation	Main Finding	Relevance and Limitation	References
Compartmental NAD^+^ availability	Engineered human cell lines with compartment-targeted NAD^+^ consumption	Subcellular NAD^+^ pools were interconnected, with mitochondrial NAD^+^ contributing to buffering of cellular NAD^+^ availability.	Supports consideration of local cofactor availability; does not directly measure SIRT1 activity or functional reserve.	[39]
Oxidative modification of SIRT1	Human airway epithelial cells exposed to oxidants and carbonyl stress	SIRT1 underwent oxidative/carbonyl modifications associated with reduced deacetylase activity, altered localization and protein degradation.	Demonstrates regulation beyond protein expression; findings depend on the oxidant and exposure conditions.	[42]
Stress-induced enhancement of SIRT1 activity	Cellular and mouse stress models; manipulation of SIRT1 O-GlcNAcylation	O-GlcNAcylation at Ser549 enhanced SIRT1 deacetylase activity and promoted cytoprotection under stress.	Shows that stress can enhance SIRT1 function; the direction of regulation depends on the modification involved.	[43]
Maintenance of the heat-shock response	Cultured cells subjected to heat shock with altered SIRT1 expression or activity	SIRT1-dependent HSF1 deacetylation prolonged HSF1 binding to the Hsp70 promoter and sustained the heat-shock response.	Supports a role in maintaining stress-response competence; response duration alone does not establish functional recovery.	[62]
Dissociation between abundance and activity	Human skeletal muscle assessed after six weeks of high-intensity interval training	SIRT1 activity increased despite a reduction in SIRT1 protein abundance.	Protein abundance alone is an incomplete indicator of SIRT1 function; training-associated changes do not establish causality.	[64]
Mitochondrial recovery after injury	Cultured renal proximal tubule cells exposed to oxidant injury and treated with SRT1720	SRT1720 promoted mitochondrial biogenesis and recovery of mitochondrial function after injury.	Supports assessment of post-stress recovery; an acute injury model does not establish restoration of aging-related adaptive capacity.	[67]
Recovery of protein synthesis	Wild-type and *SIRT1*-deficient mouse embryonic fibroblasts exposed to endoplasmic reticulum stress	SIRT1 deficiency was associated with sustained eIF2α phosphorylation and delayed recovery of protein synthesis.	Provides evidence linking SIRT1 to recovery kinetics; stress-pathway activation and successful recovery are distinct outcomes.	[23]
Nonlinear effects of SIRT1 expression	Transgenic mice with different levels of cardiac SIRT1 overexpression	Low-to-moderate overexpression protected against age-associated cardiac changes and oxidative injury, whereas high overexpression promoted oxidative stress and cardiomyopathy.	Supports dose-dependent effects; the expression levels studied do not define a universal adaptive threshold.	[74]
SIRT1 turnover during senescence	Senescent human fibroblasts and aging-associated immune/hematopoietic systems	Nuclear SIRT1 was targeted for autophagic degradation, contributing to reduced protein abundance during senescence and aging.	Identifies protein turnover as a constraint on SIRT1 availability; does not establish a universal cause of cellular senescence.	[75]
Dependence on cellular context and downstream machinery	SIRT1 overexpression in compressed human nucleus pulposus cells and irradiated BJ/TERT fibroblasts	Protection against compression-induced senescence required *PINK1*-dependent mitophagy; sustained SIRT1 overexpression did not suppress irradiation-induced SASP in BJ/TERT fibroblasts.	SIRT1 abundance is not sufficient to predict protection; downstream competence and the senescence model influence outcomes.	[76,77]

Abbreviations: eIF2α, eukaryotic translation initiation factor 2α; HSF1, heat shock factor 1; NAD^+^, oxidized nicotinamide adenine dinucleotide; O-GlcNAc, O-linked N-acetylglucosamine; PINK1, PTEN-induced kinase 1; SASP, senescence-associated secretory phenotype; SIRT1, sirtuin 1; TERT, telomerase reverse transcriptase.

### 3.2. Repeated Stress and Incomplete Recovery

Repeated stress differs from a single transient challenge because protective pathways must be repeatedly activated and subsequently return toward baseline. Adaptive homeostasis is relevant to this process because it considers both induction and subsequent de-induction of stress-protective systems [15,55]. When recovery between challenges becomes incomplete, the physiological state before a subsequent challenge may differ from the original baseline.

Recovery is an active and energetically demanding phase of the stress response. It depends on mitochondrial processing capacity, metabolic flexibility, and coordinated stress-response pathways [54,65,70]. Aging is associated with reduced adaptive flexibility and impaired adjustment of stress-protective systems [16,59,60]. SIRT1 may contribute to recovery through its interactions with metabolic and stress-response pathways, but incomplete recovery should not be interpreted as evidence of SIRT1 exhaustion.

The SIRT1–AMPK relationship illustrates this context dependence. AMPK activation can increase NAD^+^ availability and support SIRT1 activity, whereas SIRT1 can promote AMPK activation through LKB1 [63]. During repeated stress, changes in either pathway may affect the ability of cells to restore metabolic balance. This network also intersects with mTOR signaling. Under nutrient-rich conditions, mTORC1 generally promotes protein synthesis and limits autophagy, whereas nutrient restriction is associated with increased AMPK and SIRT1 activity and reduced mTORC1 signaling [23]. However, mTOR activity during stress is dynamic rather than uniformly harmful. For example, H_2_O_2_ exposure can cause an early reduction in mTOR activity followed by reactivation during autophagic lysosome reformation [78]. Nutrient-sensing pathways therefore place mTOR, AMPK, and NAD^+^-dependent sirtuins within a broader regulatory network that coordinates metabolic adaptation, autophagy, proteostasis, mitochondrial function, and stress responses [79,80,81].

The SIRT1–AMPK–mTOR network provides a broader context for these interactions. Under nutrient-rich conditions, mTORC1 is active and promotes protein synthesis while inhibiting autophagy, whereas AMPK and SIRT1 are relatively inactive. Under nutrient-restricted conditions, AMPK and SIRT1 are activated, mTORC1 is inhibited, and autophagy and mitochondrial quality control are enhanced. This coordinated response is consistent with the observation that dietary restriction engages conserved nutrient-sensing pathways, including insulin/IGF-1 signaling, mTOR, AMPK, and NAD^+^-dependent sirtuins, which converge on key transcription factors to coordinate metabolic and longevity-associated gene expression [79]. Under persistent stress, however, this network may become dysregulated, with mTOR reactivation despite continued stress, contributing to incomplete recovery and maladaptive transition [80,81].

Recovery is also evident in the heat shock response. SIRT1-dependent deacetylation of HSF1 can prolong HSF1 activity during proteotoxic stress, whereas subsequent attenuation is required for the response to return toward baseline [62,82]. Aging may impair this inducible response, with increased basal stress-protective activity accompanied by a reduced capacity for further induction [59,83]. This pattern has been described as a physiological ceiling in adaptive homeostasis [59]. It does not represent a universal threshold, but it illustrates how repeated stress may reduce response flexibility without causing immediate cellular failure. Mitochondrial stress responses are similarly influenced by stress history and cellular context and involve turnover, biogenesis, proteostasis, and redox signaling [84]. Changes in SIRT1 and SIRT3 subcellular distribution during ischemic stress in aged cardiac tissue further illustrate that stress history can influence where SIRT1 acts and how its activity is integrated with mitochondrial regulation [85].

Repeated stress therefore does not necessarily produce a progressive loss of SIRT1 activity. Instead, the cellular conditions that shape SIRT1-dependent responses may change, including NAD^+^ availability, mitochondrial function, proteostasis, and stress-responsive signaling [68,86,87,88]. Incomplete recovery may modify subsequent SIRT1 function without indicating SIRT1 exhaustion. Such changes may remain reversible when stress burden decreases and recovery mechanisms are preserved. The distinction between reversible compensation and persistent dysfunction is therefore better assessed from the trajectory of cellular function over time than from a single stress-response marker.

### 3.3. Compensatory Signaling and Diminishing Returns

When stress persists or recurs, compensatory responses become increasingly important for maintaining cellular function. Compensation itself does not necessarily indicate pathology; it can refer to a state in which additional regulatory activity or remodeling is required to maintain function as conditions increasingly challenge the original homeostatic range [16,49,89]. How SIRT1 contributes to this transition is complicated by findings that do not always point in the same direction. In the heart, for example, moderate SIRT1 overexpression increases resistance to oxidative stress and attenuates several age-associated phenotypes, whereas higher levels produce adverse cardiac effects [74]. Greater SIRT1 abundance therefore does not translate into proportionally greater protection. Tissue context adds further complexity. Caloric restriction affects SIRT1 expression and activity differently across tissues [90], and genetic studies in calorie-restricted mice suggest that SIRT1 contributes to the response without requiring a general increase in expression for lifespan extension [70]. SIRT1 also operates within a highly interconnected protein network. Multiple protein–protein interactions influence its localization, substrate engagement, stability, and catalytic output, and tissue-specific network organization can alter the consequences of SIRT1 modulation [69]. A regulatory component may therefore become limiting even when SIRT1 itself remains enzymatically active.

Protein quality control represents one such constraint, and the relationship between SIRT1 and proteostasis is bidirectional. SIRT1 contributes to proteostasis by deacetylating HSF1, thereby prolonging the heat shock response [62,91], and by promoting autophagic flux through deacetylation of key autophagy regulators [82]. Conversely, SIRT1 is itself a client of the Hsp90 chaperone system. Hsp90 stabilizes SIRT1 in mammalian cells and *C. elegans*, whereas disruption of Hsp90 promotes SIRT1 depletion and proteasomal degradation [92,93]. A feed-forward circuit linking SIRT1 and HSP90α has also been described, in which SIRT1 regulates HSP90α expression through HSF1, while HSP90α chaperones and stabilizes SIRT1 [93]. During aging, this bidirectional interaction may become compromised. Reduced proteostatic capacity could destabilize SIRT1, while reduced SIRT1 activity could further impair proteostasis. This may create a reinforcing interaction between impaired proteostasis and reduced SIRT1-dependent stress responses.

A similar principle applies to mitochondrial stress. SIRT1 influences mitochondrial biogenesis through PGC-1α and participates in mitochondrial quality-control responses, while mitochondrial metabolic state influences the environment in which SIRT1 operates [94]. The observation that Sirt1 coordinates the mitochondrial unfolded protein response and proteostasis in zebrafish muscle further supports the possibility that SIRT1-dependent adaptation requires coordinated function across multiple quality-control systems [68]. A limitation in one component may therefore constrain the benefit of modulating another.

These observations suggest that compensatory states are characterized less by absolute increases in SIRT1 activity than by increasing dependence on SIRT1-associated pathways to preserve cellular function. Further enhancement of SIRT1 activity may yield progressively smaller benefits if other determinants of adaptation become limiting. This provides a possible explanation for the diminishing returns observed in some SIRT1 manipulation studies [74]. The concept of diminishing returns should be distinguished from progressive SIRT1 failure. A cell may retain measurable SIRT1 activity while showing reduced flexibility in how that activity is deployed across changing conditions. Such a state could remain reversible if stress burden is reduced and recovery mechanisms remain functional. Conversely, if compensatory demands persist while recovery becomes increasingly incomplete, cellular function may become more dependent on sustained stress-response activity and less capable of returning to its previous functional range [16,59,89].

## 4. Maladaptive State Transition: From Compensation Failure to Persistent Dysfunction

### 4.1. Failure of Recovery Under Persistent Stress

The transition from compensatory adaptation to persistent dysfunction is unlikely to result from a single molecular event. It may occur when stress-responsive pathways remain activated while the processes required for recovery become insufficient. Persistent DNA damage, mitochondrial dysfunction, metabolic imbalance, and inflammatory signaling can each contribute to cellular senescence, but their relative contributions depend on the initiating stress and cellular context [95,96,97,98]. Cellular senescence is therefore heterogeneous rather than a uniform endpoint of a single pathway. Senescent cells can differ in metabolic state, secretory profile, and upstream stress signals, even when they share features such as stable cell-cycle arrest and persistent damage responses [95,96,99].

Persistent DNA damage is one well-characterized route to senescence. Prolonged activation of the DNA damage response engages p53–p21 and p16–RB signaling and restricts cell-cycle progression when damage cannot be resolved [97]. Oxidative stress can contribute by increasing DNA lesions and sustaining damage signaling, although ROS accumulation alone is not sufficient to define senescence [98]. Mitochondrial dysfunction provides another route. Mitochondrial impairment can promote senescence through changes in energy metabolism, NAD^+^/NADH balance, mitochondrial quality control, and stress signaling [100,101,102]. Mitochondrial dysfunction-associated senescence (MiDAS) can exhibit a secretory profile distinct from that induced by genotoxic stress and, in human cells, is associated with a reduced NAD^+^/NADH ratio and a modified SASP [103]. Thus, persistent dysfunction cannot be attributed to oxidative damage alone; metabolic and mitochondrial changes also influence how cells respond to and recover from stress.

SIRT1 participates in several of these processes, but loss of SIRT1 is not a universal prerequisite for senescence. SIRT1 regulates proteins involved in DNA damage responses, stress resistance, metabolism, and inflammatory signaling, and its abundance and activity can change during senescence [6,75,91,104]. In human fibroblasts, SIRT1 protein is reduced during several forms of senescence, and this reduction can involve autophagy-dependent degradation rather than decreased mRNA [75]. Persistent stress may therefore affect SIRT1 through changes in protein turnover and intracellular trafficking as well as through transcriptional regulation or direct inhibition of enzymatic activity. Under transient stress, changes in SIRT1 activity may be reversible and integrated into broader stress-response programs. With persistent stress, changes in NAD^+^ metabolism, mitochondrial function, protein turnover, and intracellular signaling may progressively alter the conditions in which SIRT1 operates. SIRT1 function may consequently become insufficient relative to cellular demands without being completely lost.

The relationship between redox imbalance and altered SIRT1 function becomes particularly relevant when stress is not efficiently resolved. Oxidative and carbonyl stress can modify SIRT1 directly, whereas changes in NAD^+^ metabolism, DNA damage responses, and mitochondrial function can affect it indirectly. Conversely, altered SIRT1-dependent regulation can influence antioxidant defense, mitochondrial homeostasis, inflammatory signaling, and DNA repair. The direction and magnitude of these interactions vary among cellular contexts. One experimentally supported mechanism involves stress-induced protein turnover. SIRT1 is selectively degraded through nuclear autophagy during senescence, accompanied by redistribution from the nucleus to the cytoplasm [75]. Reduced SIRT1 protein under senescent conditions may therefore reflect altered proteostasis and trafficking rather than reduced transcription. SIRT1 also suppresses NF-κB-dependent inflammatory signaling and influences senescence-associated inflammatory mediators [6,105]. In human dermal fibroblasts, pharmacological enhancement of SIRT1 activity reduced NF-κB acetylation, inflammatory cytokine expression, and several senescence-associated phenotypes [105]. Disruption of the ATM–LARP7–SIRT1 axis has been associated with increased p53 and NF-κB activity and accelerated senescence [106]. SIRT1 can also deacetylate p53 and thereby modify p53-dependent stress responses [107]. Reduced SIRT1 expression has been associated with increased p53 acetylation and activation of the DNA damage response in oxidative stress models [108]. In aging, oxidative stress-induced impairment of the Nur77–SIRT1 axis has been linked to increased DNA damage and senescence-associated phenotypes [98,108]. These findings support an interaction between persistent redox stress and altered SIRT1 regulation, but do not establish a universal positive-feedback loop.

Redox perturbations can also induce adaptive responses, and SIRT1 activity can be maintained or enhanced under some stress conditions. The consequences of altered SIRT1 signaling depend on the substrate, subcellular compartment, metabolic state, and duration of stress. The proposed relationship is therefore better viewed as a potential mechanism through which unresolved stress may become more difficult to reverse in some cellular contexts, rather than as an obligatory sequence.

Persistent stress also has extracellular consequences. Senescent cells develop a senescence-associated secretory phenotype (SASP) that includes cytokines, chemokines, growth factors, and proteases [105,109]. SASP composition varies with the initiating stimulus and metabolic environment [103,109]. Cytoplasmic chromatin fragments can activate cGAS–STING signaling and promote inflammatory gene expression [110], while mitochondrial DNA released during minority mitochondrial outer-membrane permeabilization can also activate cGAS–STING and contribute to SASP production [111]. cGAS–STING activity has been detected in aged tissues, including microglia, where mitochondrial DNA accumulation can activate inflammatory signaling [112]. These findings do not establish SIRT1 as an upstream regulator of cGAS–STING during aging. Rather, they illustrate how persistent mitochondrial and genomic stress can create an inflammatory environment that influences SIRT1-dependent responses.

Persistent redox, metabolic, mitochondrial, and genomic stress can therefore alter the cellular conditions in which SIRT1 operates. The consequences of reduced SIRT1 activity extend beyond antioxidant responses to DNA damage signaling, inflammatory regulation, mitochondrial maintenance, and proteostasis. However, the available evidence remains context-dependent, and whether these processes form a self-reinforcing loop during normal aging remains to be determined.

### 4.2. Adaptive-to-Maladaptive Transition and the Adaptive Threshold

The accumulation of persistent dysfunction does not represent a discrete molecular switch from “adaptive” to “maladaptive.” Cells exposed to chronic stress can occupy heterogeneous intermediate states characterized by different combinations of stress signaling, metabolic remodeling, damage accumulation, and recovery capacity. Cellular senescence is itself heterogeneous: p16 and p21 expression, SA-β-gal activity, and SASP components vary across cell types, tissues, and senescence-inducing conditions, and no single marker is sufficient to define senescence across biological contexts [95,96,113,114]. These states are represented in Figure 2 as conceptual regions within a continuous adaptive landscape, rather than as discrete stages.

The adaptive-to-maladaptive transition refers here to a change in the relationship between cellular demand and the capacity to maintain or restore homeostasis, rather than activation of a single senescence pathway. A cell may initially compensate for increased redox or metabolic demand by modifying antioxidant defenses, mitochondrial activity, proteostasis, or inflammatory signaling. With persistent stress, these responses may remain chronically engaged or become progressively altered. When the capacity to maintain or restore homeostasis becomes insufficient, persistent phenotypic changes can emerge, including stable cell-cycle arrest, mitochondrial dysfunction, altered proteostasis, inflammatory signaling, and changes in cellular morphology [98,103,115]. SIRT1 participates at several points along this continuum. During adaptive responses, SIRT1-dependent regulation of transcription factors and metabolic pathways can support stress tolerance and recovery. During prolonged stress, SIRT1 function can be altered by changes in NAD^+^ availability, post-translational regulation, protein stability, intracellular localization, and substrate engagement. Thus, the same level of SIRT1 protein or activity may not produce equivalent functional output under different cellular conditions. This distinction provides a basis for separating SIRT1 abundance from SIRT1 functional reserve.

Several experimental observations support this distinction. SIRT1 inhibition or depletion is associated with increased senescence-associated phenotypes in several cellular models, whereas increased SIRT1 activity or expression can attenuate senescence under specific conditions [116,117]. For example, SIRT1 depletion in human umbilical vein endothelial cells induces a premature senescence-like phenotype with increased SA-β-gal activity and sustained growth arrest [116,117]. These findings indicate that SIRT1 status can influence senescence-associated phenotypes in specific contexts, but they do not show that increasing SIRT1 is sufficient to reverse cellular aging. The effects depend on cell type, initiating stress, intervention, and metabolic state. Senescent states also differ in their redox and mitochondrial characteristics. MiDAS has a secretory profile distinct from that associated with genotoxic stress [103], and oxidative stress-induced DNA damage can engage cGAS–STING–NF-κB signaling [118]. The composition and magnitude of the SASP likewise vary with the initiating stimulus and cellular context [96,119]. Two cells with similar SIRT1 levels may therefore show different functional phenotypes if they differ in mitochondrial status, DNA damage burden, metabolic substrate availability, or inflammatory signaling.

This context dependence is also evident in SIRT1 manipulation studies. When NAD^+^ availability and mitochondrial function are relatively preserved, increasing SIRT1 activity may support responses to reversible stress. When persistent mitochondrial dysfunction, DNA damage, or impaired proteostasis limits downstream processes, increasing SIRT1 activity alone may have more limited effects. SIRT1 overexpression attenuates compression-induced senescence and mitochondrial dysfunction in human nucleus pulposus cells through PINK1-dependent mitophagy [76], whereas enforced SIRT1 expression does not suppress the SASP during senescence induction in human fibroblasts [77]. These contrasting findings indicate that the consequences of SIRT1 modulation depend on cellular context and the nature of the accompanying stress rather than supporting a universal anti-aging effect [120].

The adaptive-to-maladaptive transition is therefore characterized by reduced reversibility and increasing dependence on compensatory mechanisms rather than by a simple decrease in SIRT1. Persistent dysfunction emerges when multiple regulatory systems can no longer efficiently restore the previous cellular state. The adaptive threshold is used here as a conceptual construct describing this change in the balance between stress burden and recovery capacity. It is not a defined numerical value and is unlikely to correspond to a single molecular concentration or activity level. Instead, the position of this threshold may reflect the combined effects of NAD^+^ availability, SIRT1 catalytic activity, mitochondrial function, redox buffering, DNA repair capacity, proteostasis, inflammatory signaling, and other determinants of recovery [96,121,122,123,124]. Its position is also expected to vary among cells. Cell type, metabolic state, mitochondrial function, proliferative history, and prior stress exposure influence responses to subsequent perturbations, while single-cell analyses indicate that senescent cells can occupy distinct transcriptional states following the same stress exposure [119,125]. Senescence induced by different stimuli and in different cell types likewise exhibits distinct molecular and epigenetic features [126].

Stress history is therefore an important component of the proposed model. Two cells exposed to the same acute oxidative challenge may respond differently if one has previously experienced repeated stress, because prior challenges can alter NAD^+^ metabolism, protein quality control, mitochondrial function, inflammatory tone, or chromatin organization. Exceeding the proposed adaptive threshold should not be equated with irreversible senescence. The probability of irreversible commitment to senescence increases with the duration of stress exposure rather than occurring at a single fixed time point [127], and some cells can regain proliferative capacity under specific experimental conditions [128,129]. The threshold therefore represents a change in cellular behavior rather than a binary molecular switch. A parallel framework for post-mitotic neurons, in which proliferative arrest cannot serve as a readout, is discussed in Section 5.4.

Within this framework, SIRT1 functional reserve represents one component of the broader adaptive capacity of the cell. It does not determine the threshold independently but interacts with NAD^+^ metabolism, mitochondrial quality control, DNA repair, proteostasis, and inflammatory signaling. Reduced SIRT1 activity alone may not be sufficient to establish a maladaptive state, and preserved SIRT1 activity may not guarantee successful recovery when other systems are severely compromised. Representative experimental evidence is summarized in Table 2.

## 5. Cellular and Tissue Contexts of SIRT1–Redox Adaptation

The adaptive and maladaptive states described above are not uniform across cells and tissues. Cellular identity, metabolic demand, mitochondrial function, prior stress exposure, subcellular localization, and local NAD^+^ availability can all influence the functional consequences of SIRT1–redox interactions. Similar changes in SIRT1 activity or redox state may therefore produce different outcomes in different cellular environments. This variability is also evident across experimental models. SIRT1 regulation differs among human cells, mouse tissues, zebrafish, and *C. elegans*, limiting direct extrapolation between systems. Table 3 summarizes representative differences across species, tissues, and stress paradigms and distinguishes broadly conserved mechanisms from model-specific observations.

### 5.1. Cell-Type-Specific Redox Demands and SIRT1 Responses

Cellular populations differ in metabolic requirements, proliferative capacity, mitochondrial activity, antioxidant systems, and exposure to stressors [134,135,136,137]. These differences influence both the magnitude of redox perturbation and the mechanisms available for recovery. Consequently, similar changes in SIRT1 abundance or activity may have different functional consequences across cell types.

Endothelial cells provide one example. They are exposed to fluctuations in oxygen tension, shear stress, inflammatory mediators, and metabolic substrates, and oxidative stress can disrupt nitric oxide signaling and vascular homeostasis [138,139]. SIRT1 participates in endothelial responses to oxidative stress, inflammation, and premature senescence [138,139,140]. In human endothelial cells, SIRT1 inhibition or depletion is associated with senescence-like phenotypes, whereas increased SIRT1 activity can attenuate selected stress-associated changes [116,141,142]. These findings indicate that SIRT1 activity can influence endothelial stress responses, but do not establish a uniform effect across vascular conditions.

Metabolically active and differentiating cells provide another example. During adipogenic differentiation, mesenchymal stromal cells undergo substantial metabolic remodeling, and changes in ROS levels can influence lineage commitment [143,144]. Oxidative stress and altered SIRT1 signaling have been associated with changes in adipogenic differentiation and senescence-associated phenotypes in mesenchymal stem cells [143,145]. SIRT1-dependent regulation of FOXO3 and SUV39H1 has also been implicated in these responses [146,147,148]. The effects of SIRT1 modulation may therefore vary with cellular differentiation state as well as with the nature of the metabolic or oxidative challenge.

Stem and progenitor cells can respond differently to aging-associated stress. In neural stem cells from the subventricular zone, aging is associated with increased Sirt1 expression, reduced DNA double-strand breaks, and enhanced non-homologous end joining-mediated repair. *Sirt1* deficiency abolishes this reduction in DNA damage and is associated with stem-cell exhaustion and impaired olfactory memory [149]. These findings indicate that SIRT1 does not necessarily decline in every aging cell population. In some cells, increased SIRT1 expression may accompany an adaptive response to increased stress or DNA damage.

Immune and glial cells provide a further example of cell-specific regulation. In aged rhesus macaque brains, microglial activation and SIRT1-related chromatin regulation differ between animals with shorter and longer survival, together with changes in CD38 and miR-142 [150,151,152,153]. In models of postoperative cognitive dysfunction and optic nerve injury, SIRT1 activation reduces microglia-mediated synaptic engulfment and synapse loss [154,155]. These findings indicate that SIRT1-dependent regulation in non-neuronal cells can influence neuronal function indirectly through inflammatory and tissue-level mechanisms.

**Figure 3:**
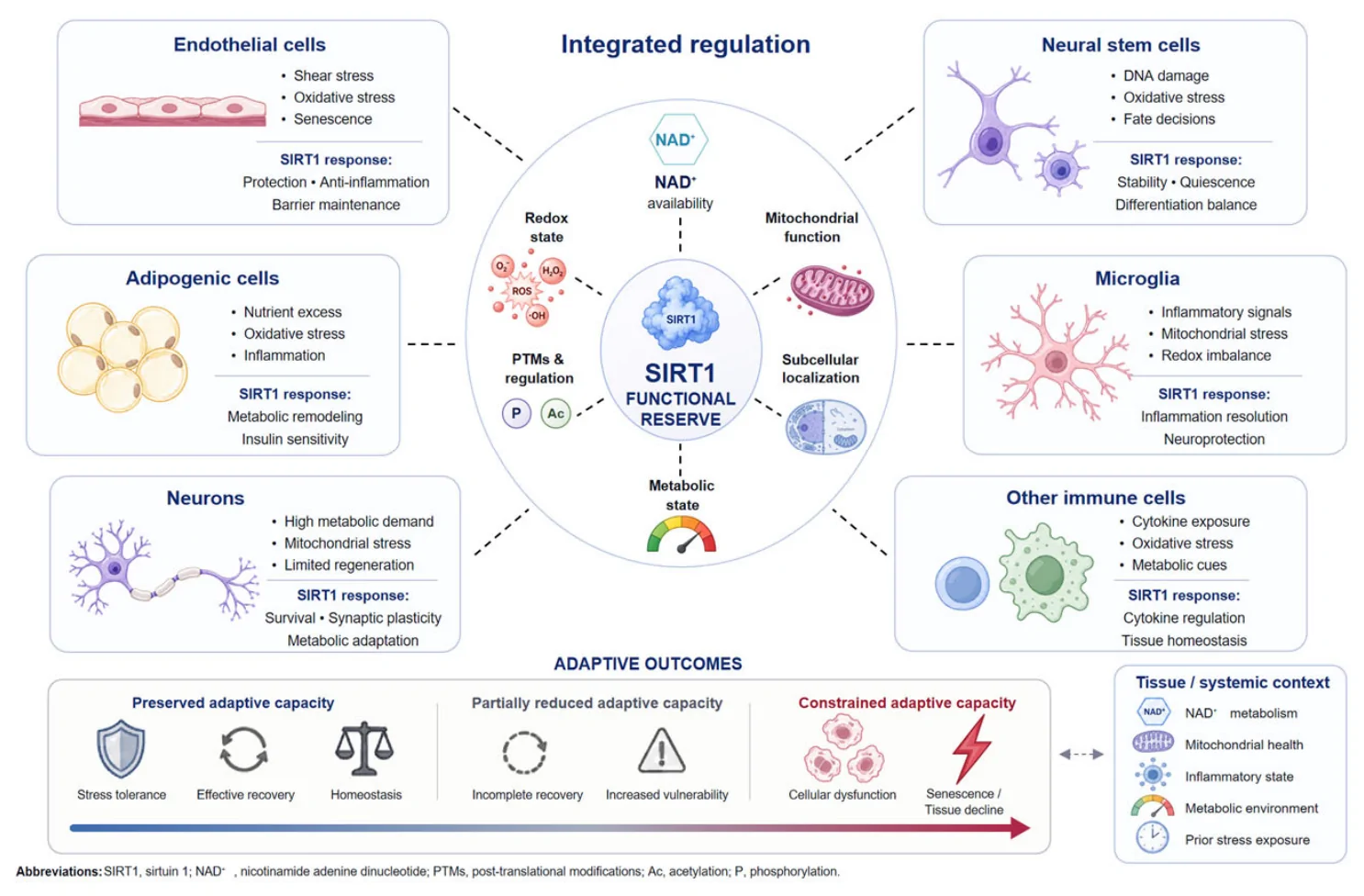
Cell- and tissue-context dependence of SIRT1 functional capacity and adaptive outcomes during aging.

Cellular identity therefore affects how SIRT1 perturbation is interpreted. Differences in metabolic demand, differentiation state, DNA repair capacity, inflammatory activity, and mitochondrial dependence influence the pathways through which SIRT1 contributes to stress adaptation [156,157,158,159]. Reduced SIRT1 activity is associated with impaired recovery in some contexts, whereas increased or redistributed SIRT1 can accompany adaptive responses in others [149,160,161]. SIRT1 regulation during aging should therefore be interpreted in relation to cell type, tissue environment, and stress history rather than as a uniform age-associated increase or decrease. Representative cell- and tissue-specific differences are summarized in Figure 3.

### 5.2. Subcellular Compartmentalization of SIRT1-Dependent Adaptation

SIRT1 function is influenced by its subcellular localization. SIRT1 is present in both the nucleus and cytoplasm, and its distribution varies among cell types and tissues [44,162,163,164]. Total cellular SIRT1 abundance therefore does not necessarily reflect the pool available to a particular substrate.

Localization is functionally relevant because SIRT1 substrates are spatially organized. Nuclear SIRT1 regulates chromatin-associated proteins and transcription factors involved in DNA damage responses, metabolism, and stress signaling, whereas cytoplasmic SIRT1 interacts with proteins involved in cytoplasmic signaling and metabolic regulation [163]. Changes in SIRT1 localization can therefore alter substrate accessibility without changing total protein abundance. In cardiac tissue, aging alters the subcellular distribution of SIRT1 and SIRT3, while stress-induced redistribution is associated with changes in mitochondrial and cardiometabolic homeostasis [85]. Nuclear and cytoplasmic SIRT1 can also produce distinct effects on p53-dependent signaling [165]. Thus, redistribution of SIRT1 may contribute to adaptation during acute stress, whereas persistent changes in localization may alter functional output.

NAD^+^ is similarly organized into distinct subcellular pools. Mitochondrial NAD^+^ availability can be functionally limiting even when total tissue NAD^+^ remains relatively preserved [39,166]. Because SIRT1 activity depends on NAD^+^, the local NAD^+^ environment may therefore influence SIRT1 function differently across compartments. Bulk NAD^+^ measurements may consequently provide an incomplete estimate of the catalytic environment experienced by SIRT1.

Substrate accessibility provides an additional level of regulation. Post-translational modification of SIRT1 can alter its interactions with regulatory proteins and influence substrate engagement and deacetylase activity [167]. SIRT1 can also interact with telomerase reverse transcriptase (TERT) and promote its nuclear localization and stability independently of deacetylase activity [168]. Thus, some SIRT1-dependent effects involve protein–protein interactions in addition to catalytic deacetylation. Together, these observations support a distinction between total SIRT1 abundance and SIRT1 functional state. Two cells with similar total SIRT1 and NAD^+^ levels may nevertheless exhibit different SIRT1-dependent responses if enzyme localization, substrate accessibility, or local cofactor availability differs. This spatial organization may become particularly relevant during aging, when changes in organelle function, protein trafficking, and metabolic compartmentalization can alter the environment in which SIRT1 operates. Compartment-specific measurements of SIRT1 abundance, catalytic activity, NAD^+^ availability, substrate engagement, and localization therefore provide experimentally accessible parameters for testing whether spatial organization contributes to differences in stress adaptation and recovery.

### 5.3. Tissue-Specific Adaptive Capacity During Aging

Cellular differences are amplified at the tissue level. Aging affects tissues according to their metabolic requirements, regenerative capacity, exposure to physiological stress, and ability to replace damaged cells [63,169,170]. These differences influence both basal SIRT1-dependent signaling and the capacity to compensate for persistent metabolic or redox perturbations.

Skeletal muscle depends on high mitochondrial activity, metabolic flexibility, and repeated adaptation to energetic demand. Aging alters skeletal muscle metabolism, mitochondrial function, proteostasis, and regenerative capacity [171,172]. Transcriptomic studies further show that aging reshapes gene-expression programs in skeletal muscle, whereas caloric restriction modifies multiple age-associated pathways [173]. Importantly, SIRT1 overexpression does not reproduce all beneficial effects of caloric restriction, indicating that SIRT1 activation alone is insufficient to recapitulate the broader adaptive response to nutritional stress [170,174]. Exercise training also engages coordinated SIRT1-, AMPK-, and PGC-1α-associated responses in skeletal muscle [175]. These responses depend on exercise intensity, nutritional state, age, and recovery conditions. Physiological activation of SIRT1 in muscle should therefore not be assumed to produce effects equivalent to pharmacological activation under persistent pathological stress.

Cardiac tissue has high metabolic demands and limited regenerative capacity. Aging alters mitochondrial quality control, proteostasis, and redox homeostasis in the heart, accompanied by activation of mitochondrial and endoplasmic reticulum stress responses [176]. SIRT1 influences cardiac mitochondrial bioenergetics and quality-control processes; SIRT1 overexpression improves mitochondrial respiration and ATP production in a model of lamin A/C deficiency-induced cardiac dysfunction [177]. In aged hearts, mitochondrial SIRT3-dependent regulation of SOD2 is also linked to oxidative stress and cardiac remodeling [178]. These observations suggest that cardiac adaptive responses depend not only on the abundance of individual sirtuins but also on their localization and integration with compartment-specific mitochondrial pathways.

The liver provides a complementary example of tissue-specific SIRT1 regulation. In aged mice subjected to liver injury, impaired hepatic SIRT1 is accompanied by increased NLRP3 inflammasome activity, inflammatory signaling, and persistent fibrosis [131]. Reduced hepatocyte SIRT1 is associated with increased HMGB1 acetylation and cytoplasmic translocation, LSEC dysfunction, and hepatic stellate cell activation, whereas SIRT1 overexpression attenuates liver injury and fibrosis [179]. Pharmacological inhibition of NLRP3 with MCC950 reduces hepatic inflammation and fibrotic remodeling in aged mice [180]. These findings indicate that SIRT1 may influence tissue recovery through inflammatory regulation in addition to redox control.

Adipose tissue provides another example of context dependence. In adipose-derived stem cells from women of different ages, aging is associated with increased mitochondrial ROS, reduced SIRT1 expression, and impaired adipogenic capacity, whereas adipogenic induction increases SIRT1 and SIRT3 expression [132]. Altered ROS–SIRT1 signaling is also linked to adipogenic differentiation in mesenchymal stem cells [145]. More broadly, recent studies identify tissue-specific differences in SIRT1 expression and metabolic signaling across brain, liver, and skeletal muscle during aging and metabolic intervention [181]. In skeletal muscle, SIRT1 overexpression in mesenchymal stem cells reduces cellular senescence, alters p53 and p65 acetylation, and enhances muscle regeneration [182].

Taken together, these observations do not support a uniform tissue-wide pattern of SIRT1 dysfunction during aging. Rather, the functional consequences of SIRT1 modulation depend on tissue-specific metabolic demand, stress burden, regenerative capacity, and the availability of downstream adaptive pathways. Systemic measurements of SIRT1 abundance or NAD^+^ concentration may therefore provide limited information about the adaptive state of an individual tissue. Tissue- and cell-specific measurements are needed to determine whether SIRT1-dependent functions remain adequately supported under defined aging and stress conditions.

### 5.4. Neuronal Vulnerability and the Post-Mitotic Framework

The nervous system provides an informative context for examining SIRT1-dependent adaptive capacity because mature neurons combine high metabolic demands, extensive mitochondrial dependence, long lifespans, and limited regenerative potential [183,184,185]. Maintaining neuronal function requires continuous ATP production, efficient proteostasis, axonal transport, and tightly controlled redox signaling. As a result, incomplete recovery from repeated metabolic or oxidative challenges may have greater consequences in neurons than in many other cell types. Neuronal vulnerability may therefore arise when persistent mitochondrial dysfunction, redox imbalance, or inflammatory stress exceeds the capacity of neurons to maintain and restore functional homeostasis [186,187,188]. In this regard, post-mitotic neurons provide a relevant context for examining how impaired recovery capacity contributes to the transition from adaptive regulation toward functional decline.

SIRT1 is expressed in neurons and participates in multiple stress-responsive pathways. For example, SIRT1–PGC-1α signaling contributes to mitochondrial biogenesis in dopaminergic neurons [189,190]. In human neural stem cells, amyloid-β-induced senescence-like changes are associated with reduced SIRT1, PGC-1α, and FOXO3 expression, whereas SIRT1 overexpression partially attenuates these alterations [130]. In addition, modulation of SIRT1/PGC-1α signaling has been linked to changes in mitochondrial dynamics, oxidative stress responses, and neuroinflammatory processes [191]. These studies suggest that neuronal SIRT1 signaling is influenced by cellular context and metabolic conditions rather than functioning as a uniformly protective pathway.

The spatial organization of SIRT1 signaling may further contribute to this context dependency. SIRT1 is present in different neuronal compartments, including the nucleus and mitochondria [192], and SIRT1-dependent regulation has been associated with mitochondrial responses following cerebral ischemia/reperfusion, including interactions with mitochondrial SIRT3 [193]. In adult neural stem cells, SIRT1 deficiency affects DNA damage response-related alternative splicing and impairs proliferation and DNA repair processes [194]. Together, these observations indicate that the consequences of altered SIRT1 signaling depend on cellular state, subcellular localization, and the specific stress context. Therefore, reduced SIRT1 abundance alone may not be sufficient to indicate impaired neuronal adaptive capacity.

Neuronal function is also shaped by interactions between neurons and glial cells. Aging is accompanied by alterations in microglial states and inflammatory signaling, which can influence neuronal circuits and cognitive function [195]. Microglial SIRT1 deficiency enhances IL-1β-associated inflammatory responses and contributes to cognitive decline in aging and neurodegenerative conditions [133]. Conversely, SIRT1 modulation may affect microglial phenotypes and inflammatory responses through mechanisms involving Drp1 phosphorylation and mitochondrial regulation [196]. These findings highlight that neuronal SIRT1-dependent regulation occurs within a broader cellular environment, where changes in glial metabolism and inflammation may indirectly influence neuronal stress responses [197,198]. However, reduced SIRT1 activity alone cannot fully explain neuronal aging or neurodegeneration.

Importantly, neuronal aging should not be considered equivalent to canonical cellular senescence. Mature neurons are predominantly post-mitotic, and their age-associated dysfunction cannot be explained by proliferative arrest. Instead, neuronal aging involves alterations in genome maintenance, mitochondrial homeostasis, proteostasis, synaptic function, and inflammatory regulation, with different neuronal populations showing distinct patterns of vulnerability [186,199]. Although senescence-like features have been reported in subsets of post-mitotic neurons under pathological conditions [200], functional recovery rather than proliferative capacity may provide a more appropriate measure of neuronal adaptive status. Relevant indicators include preservation of synaptic plasticity, axonal transport, mitochondrial quality control, proteostasis, and redox balance.

From the perspective of SIRT1 functional reserve, transient stress may activate SIRT1-dependent responses involving FOXO3, PGC-1α, and HSF1, thereby supporting mitochondrial maintenance, antioxidant defense, and proteostatic adaptation [62,82]. However, repeated or persistent stress may progressively increase the demand placed on these systems. When NAD^+^ availability becomes limited, mitochondrial function declines, or proteostatic and redox control become impaired, neurons may lose the ability to efficiently restore homeostasis after subsequent challenges. Thus, in post-mitotic neurons, the transition toward maladaptation is better characterized by reduced reversibility and progressive functional impairment rather than by cell-cycle arrest. This interpretation places neuronal aging within the proposed SIRT1 functional reserve framework, where the critical factor is not simply whether SIRT1 signaling is activated, but whether sufficient regulatory capacity remains to support recovery after stress.

Neuronal SIRT1 functional reserve is therefore likely determined by the interaction among NAD^+^ metabolism, mitochondrial integrity, proteostasis, and redox state. Neuronal NAD^+^ pools are spatially regulated, and mitochondrial NAD^+^ availability may become limiting even when total cellular NAD^+^ levels are relatively preserved [39,166]. SIRT1 supports mitochondrial biogenesis through PGC-1α, although this function depends on the surrounding metabolic environment and mitochondrial status [201,202]. In parallel, SIRT1 contributes to stress-responsive protein quality control through HSF1 deacetylation and autophagic regulation [62,82,83], whereas impaired proteostasis may further disrupt SIRT1-dependent regulation. Persistent oxidative stress may also modify SIRT1 and alter the cellular environment in which it functions. These interconnected constraints provide a basis for investigating why neuronal SIRT1 responses vary across aging stages, neuronal populations, and stress conditions.

Overall, the neuronal context emphasizes an important aspect of the proposed framework: changes in neuronal adaptive capacity should not be inferred from a single measurement of SIRT1 abundance or activity. Instead, neuronal SIRT1 functional reserve should be evaluated through the coordinated relationship among NAD^+^ availability, SIRT1-dependent signaling, mitochondrial and proteostatic status, redox burden, and recovery of neuronal function after defined challenges. For post-mitotic cells, preservation of function and restoration capacity may therefore provide more informative measures of adaptive capacity than proliferative competence.

## 6. Therapeutic Implications: Restoring Adaptive Capacity Rather than Simply Activating SIRT1

The context dependence of SIRT1–redox interactions has important therapeutic implications. If SIRT1-dependent responses are limited by NAD^+^ availability, redox state, metabolic conditions, subcellular organization, or persistent cellular dysfunction, increasing SIRT1 activity alone may have limited effects. Therapeutic strategies may instead need to address the conditions that support SIRT1 function while reducing persistent stress. SIRT1 activation, NAD^+^ restoration, mitochondrial and redox modulation, and interventions targeting senescence-associated dysfunction can therefore be considered as complementary approaches rather than interchangeable strategies.

### 6.1. Limitations of SIRT1 Activation Alone

Pharmacological SIRT1 activation can modify metabolic and stress-responsive pathways, but its translation to human aging remains limited. In a randomized phase I study in older adults, the selective SIRT1 activator SRT2104 was well tolerated and produced changes in lipid profiles and trends toward faster phosphocreatine and ADP recovery after exercise, but broad metabolic or physiological benefits were not demonstrated [203]. A randomized crossover study in patients with type 2 diabetes also reported cardiometabolic effects without consistent improvement in glycemic control [204]. These studies show that pharmacological target engagement does not necessarily translate into a uniform functional benefit. Reviews of SRT2104 development have similarly emphasized the importance of disease context and the biological processes being targeted [205].

One possible explanation is that SIRT1 activity depends on the cellular environment. NAD^+^ availability, substrate accessibility, subcellular localization, mitochondrial function, and downstream stress-response pathways can all influence the consequences of SIRT1 activation. Increasing catalytic activity may therefore have limited effects when one or more of these components remains constrained. Pharmacological activation may also affect only selected SIRT1-dependent functions and may not reproduce the spatial and temporal regulation of endogenous SIRT1 signaling. These considerations argue for evaluating SIRT1 activation together with the cellular conditions that determine its functional output.

### 6.2. Restoring NAD^+^ Availability

Because NAD^+^ availability constrains SIRT1 activity, restoring NAD^+^ metabolism represents a potential complementary approach. Human studies, however, have produced mixed functional outcomes. In older adults with mild cognitive impairment, nicotinamide riboside (NR) increased blood NAD^+^ approximately 2.6-fold over 10 weeks but did not improve the primary cognitive outcome or most secondary functional measures [206]. In a separate randomized placebo-controlled study, 12 weeks of nicotinamide mononucleotide (NMN) supplementation increased blood NAD^+^ and was associated with modest changes in selected walking and sleep measures, whereas the primary stepping-test outcome did not differ significantly between groups [207]. Thus, biochemical target engagement does not necessarily translate into functional improvement.

This distinction is also evident in clinical reviews. Vinten et al. concluded that evidence for age-related NAD^+^ decline in humans remains dependent on the tissue examined and the measurement method used, while clinical studies have generally reported only limited and variable functional benefits [41]. Consistent with this, a 2026 systematic review of 33 human intervention studies found relatively consistent increases in circulating or cellular NAD-related metabolites after oral NR or NMN, but inconsistent or often null effects on functional, metabolic, and vascular outcomes [208]. Increasing systemic NAD^+^ should therefore not be assumed to restore SIRT1-dependent function in every tissue.

NAD^+^ metabolism is regulated by multiple biosynthetic and consuming pathways, and responses to precursor supplementation depend on tissue uptake, metabolic conversion, NAD^+^ consumption, and intracellular compartmentalization. Clinical responses may also vary with metabolic and disease status. In a pilot randomized trial of middle-aged and older adults with hypertension, NR combined with exercise did not reduce daytime systolic blood pressure more than exercise alone, despite increases in NAD-related metabolites [209]. In older adults with peripheral artery disease, a pilot study of NR reported biochemical and vascular effects that require further evaluation in larger controlled studies [210]. These findings suggest that circulating NAD^+^ concentration alone is unlikely to capture the functional consequences of NAD^+^ supplementation. Measurements of tissue-specific NAD^+^ metabolism, SIRT1 activity, substrate deacetylation, and functional recovery may provide additional information.

### 6.3. Modulating Mitochondrial and Redox Stress

Another approach is to modify the cellular stress environment in which SIRT1 operates. Mitochondria are both sources and targets of redox signaling. Excessive mitochondrial oxidant production can contribute to persistent dysfunction, whereas transient redox signals participate in physiological adaptation. Therapeutic strategies should therefore distinguish sustained or excessive oxidative stress from redox signaling required for adaptive responses.

Human studies illustrate the difficulty of modifying mitochondrial oxidant production without improving broader cellular function. In a randomized, double-blind, placebo-controlled trial in adults aged 65–80 years, 12 weeks of the mitochondria-targeted antioxidant MitoQ reduced mitochondrial H_2_O_2_ emission capacity in skeletal muscle but did not improve mitochondrial respiration or several measures of exercise-induced redox signaling [211]. Lowering mitochondrial oxidant production therefore did not produce a corresponding improvement in mitochondrial bioenergetics or exercise-related redox responses.

These findings are relevant to SIRT1 because mitochondrial dysfunction can alter NAD^+^ metabolism, redox signaling, and substrate availability. Correcting mitochondrial dysfunction may consequently improve the conditions in which SIRT1 operates. Conversely, excessive suppression of ROS could interfere with redox signals involved in adaptation. The therapeutic objective should therefore be restoration of an appropriate redox environment rather than maximal suppression of oxidative signals.

### 6.4. Targeting Persistent Maladaptive Cellular States

When cellular stress progresses to persistent senescence-associated dysfunction, restoring adaptive signaling alone may be insufficient. Senolytic and senomorphic approaches target these persistent cellular states [212,213], but clinical responses remain heterogeneous and may depend on the burden and characteristics of senescent cells. NAD^+^ restoration may be more relevant when adaptive responses remain partially preserved, whereas interventions targeting senescence-associated dysfunction may become more relevant when persistent dysfunction has been established.

### 6.5. Toward Context-Dependent SIRT1 Modulation

The clinical evidence does not support a simple relationship between greater SIRT1 activity and better functional outcomes. Direct SIRT1 activation, NAD^+^ restoration, mitochondrial redox modulation, and senescence-targeting interventions address different biological constraints and may act differently across tissues and disease states. The relevant therapeutic question is therefore not simply whether SIRT1 should be activated, but which factor currently limits SIRT1-dependent responses.

The adaptive-capacity framework proposed here remains conceptual and has not been prospectively validated. Current clinical studies do not establish that stratifying patients according to SIRT1 functional reserve or related biomarkers would improve treatment outcomes. Biomarker-based stratification should therefore be regarded as a direction for future investigation rather than an established clinical strategy.

Importantly, the proposed framework is not intended to suggest that SIRT1 functional reserve can currently serve as a clinical predictor. Rather, it provides a conceptual explanation for why individuals with similar levels of SIRT1 activation or NAD^+^ restoration may exhibit different biological responses. Future studies should determine whether integrated assessment of NAD^+^ metabolism, mitochondrial function, inflammatory status, and SIRT1-dependent signaling can better characterize adaptive capacity and identify biological conditions associated with differential responses to SIRT1-targeted interventions.

Several candidate biomarkers could be examined in future studies. Tissue-specific NAD^+^ availability, measured in accessible tissues such as peripheral blood mononuclear cells (PBMCs), may provide information beyond circulating NAD^+^ concentration. In a post hoc analysis of a double-blinded clinical trial in healthy adults aged 40–65 years, the increase in blood NAD^+^ after NMN supplementation showed substantial inter-individual variation, with a coefficient of variation of 29.2–113.3%, suggesting that NAD^+^ responses may differ considerably between individuals [214]. SIRT1 catalytic activity in PBMCs has also been proposed as a surrogate measure of SIRT1 functional state, while circulating miR-34a may provide an additional candidate marker [215]. Senescence-associated markers such as p16^INK4a^ in peripheral blood T cells may help characterize senescent-cell burden. In the phase II D+Q trial, responses in skeletal measures were stronger among participants with higher markers of senescent-cell burden [212]. Composite approaches incorporating mitochondrial DNA copy number, circulating microRNAs, and inflammatory markers have also been proposed for future patient stratification [216,217].

These measurements may help distinguish different biological constraints. For example, low NAD^+^ availability with preserved SIRT1 protein and downstream responsiveness would represent a different state from persistent mitochondrial dysfunction, impaired proteostasis, or established senescence-associated dysfunction. However, whether such profiles can predict treatment response remains unknown. Recent clinical studies of NAD^+^ precursors and senolytic interventions illustrate this uncertainty, as biochemical or cellular target engagement has not consistently produced equivalent functional effects across study populations [208,212,213].

**Figure 4:**
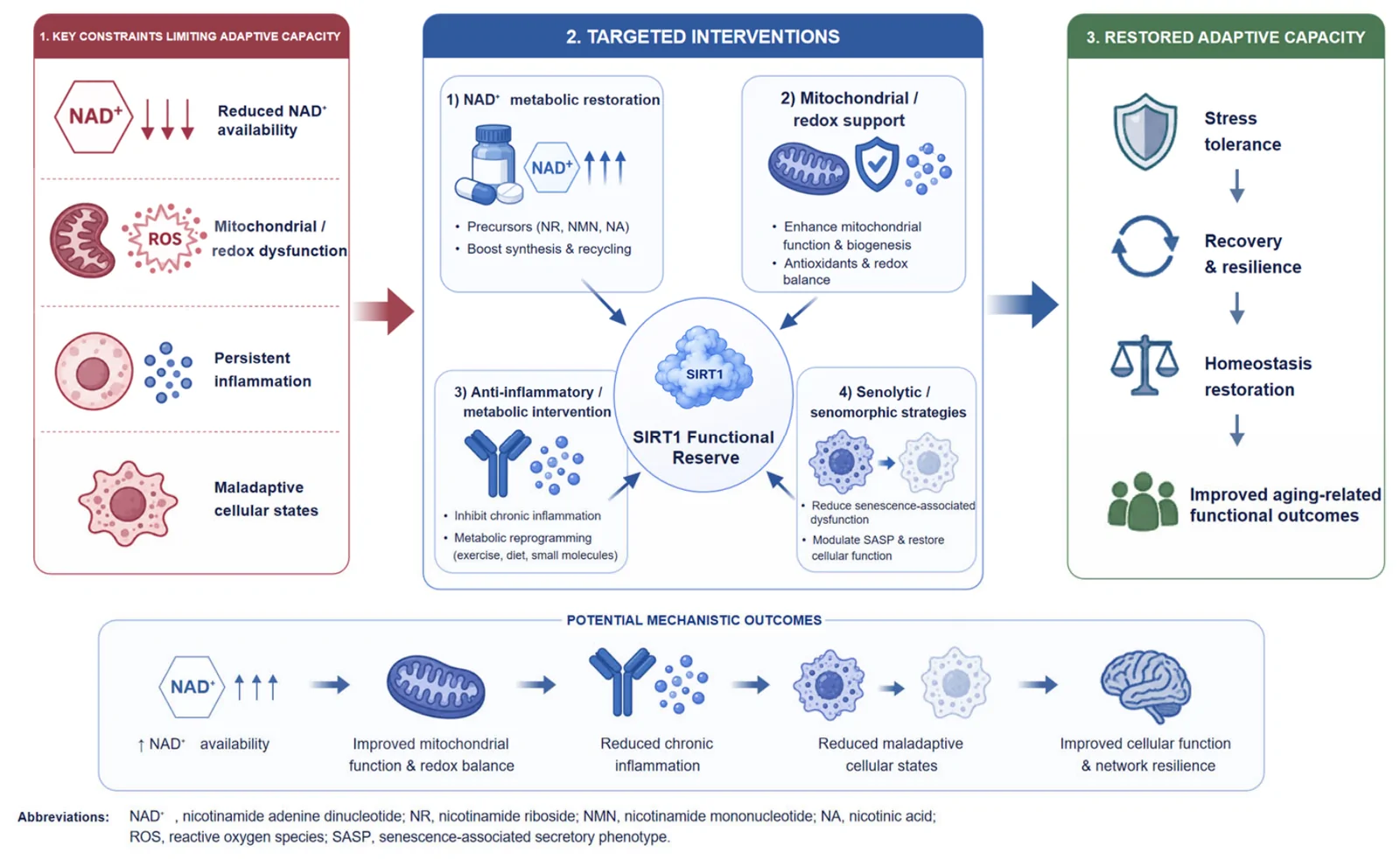
Therapeutic framework for restoring SIRT1-dependent adaptive capacity by targeting key constraints during aging.

The therapeutic implication is therefore to consider SIRT1 within the cellular environment in which it functions. In cells with preserved adaptive capacity, interventions targeting NAD^+^ metabolism or metabolic and mitochondrial stress may improve the response to subsequent challenges. When persistent dysfunction or senescence-associated phenotypes dominate, additional interventions may be required. This distinction provides a testable basis for future studies of SIRT1–redox biology in aging and age-related disease. Figure 4 summarizes the proposed therapeutic framework, and selected clinical studies are listed in Table 4.

## 7. Conclusions and Future Directions

### 7.1. Conclusions

SIRT1–redox interactions have often been described as a linear pathway in which oxidative stress reduces SIRT1 activity and impaired SIRT1 function contributes to cellular dysfunction. Experimental evidence supports several components of this model, but the relationship between SIRT1 and redox regulation is not unidirectional. SIRT1 function depends on NAD^+^ availability, post-translational regulation, subcellular localization, substrate accessibility, metabolic state, and the intensity and duration of stress. At the same time, reactive species can act as signaling molecules or contribute to molecular damage depending on their concentration, localization, chemical properties, and persistence. These factors make a simple oxidative stress–SIRT1 axis insufficient for describing SIRT1 biology during aging.

In this review, SIRT1–redox crosstalk is considered within a broader cellular stress-adaptation system. Adaptive capacity refers here to the ability of a cell to initiate, coordinate, and recover from stress responses while maintaining or restoring cellular function. This concept overlaps with, but is distinct from, adaptive homeostasis and cellular resilience. It is not intended as a single biological variable. Rather, it encompasses several processes, including redox buffering, NAD^+^ metabolism, mitochondrial function, proteostasis, DNA repair, inflammatory signaling, and recovery kinetics. Within this framework, SIRT1 functional reserve describes the remaining capacity of SIRT1-dependent regulatory processes to respond to a defined cellular demand. This distinction is important because SIRT1 protein abundance does not necessarily reflect functional capacity. NAD^+^ availability, intracellular localization, substrate accessibility, and post-translational regulation can all constrain SIRT1-dependent responses. Conversely, compensatory mechanisms may preserve particular SIRT1-dependent functions without increasing total SIRT1 abundance.

The adaptive landscape proposed in this review is intended to represent a range of cellular states rather than a fixed sequence of molecular events. Transient stress can be accommodated by reversible changes in metabolism, redox signaling, proteostasis, and transcription. With repeated or persistent stress, compensatory responses may become increasingly important. When recovery is insufficient, persistent dysfunction and senescence-associated phenotypes may emerge. The timing and extent of these changes are unlikely to be defined by a single molecular threshold and may differ substantially among cell types and tissues. Single-cell studies further indicate that senescence comprises heterogeneous cellular states and trajectories rather than a uniform endpoint [119,139]. This heterogeneity may contribute to differences in responses to senolytic interventions, although direct evidence linking senescence heterogeneity to SIRT1 functional reserve remains limited.

The evidence reviewed here supports a shift away from viewing SIRT1 activation as intrinsically beneficial. During aging, changes in NAD^+^ metabolism, mitochondrial function, proteostasis, inflammatory signaling, and cellular senescence occur simultaneously and may influence the outcome of SIRT1 modulation. Recent studies of NAD^+^ biology also emphasize tissue specificity and subcellular compartmentalization, indicating that circulating NAD^+^ levels cannot be assumed to represent SIRT1-dependent function in individual tissues [33,140]. Thus, the biological effect of SIRT1 modulation is likely to depend on whether the cellular environment can support an appropriate SIRT1-dependent response.

### 7.2. Experimental Priorities: Operationalizing SIRT1 Functional Reserve

A major challenge is to determine whether SIRT1 functional reserve can be converted from a conceptual construct into measurable experimental parameters. We therefore propose a defined stress-and-recovery protocol (Figure 1) that integrates measurements of SIRT1 function, NAD^+^ availability, redox state, mitochondrial function, stress responses, and recovery kinetics. The proposed parameters and time points are based on experimental approaches reported in the literature and are summarized in Table 1.

Primary human diploid fibroblast TIG-3 cells provide one possible model for studying SIRT1–redox responses. H_2_O_2_ exposure in these cells has been associated with NAD^+^ depletion, increased p53 acetylation, increased p21 expression, cell-cycle arrest, and increased SA-β-gal activity [17]. Mouse embryonic fibroblasts (MEFs) exposed to sublethal H_2_O_2_ have also been used to examine SIRT1 activity and p53 acetylation during subsequent recovery [215]. For neuronal studies, human iPSC-derived retinal ganglion cells exposed to tert-butyl hydroperoxide (TBHP) provide an additional model for examining SIRT1-dependent responses to oxidative stress [218]. These models differ in species, cellular state, and biological context and should therefore be viewed as complementary rather than interchangeable.

Two stress paradigms can be used to examine different aspects of adaptive capacity. The first is a controlled oxidative challenge using H_2_O_2_ at approximately 150–500 μM for 30 min to 2 h, as reported in previous cellular studies [17,219]. The second is a sublethal mitochondrial challenge using rotenone at approximately 10–500 nM, which can be used to examine responses to complex I inhibition and mitochondrial stress [220,221]. The exact concentration and exposure time should be established empirically for each cell model to ensure that the challenge produces measurable stress without causing extensive cell death.

Measurements should be collected before stress, immediately after the challenge, and during recovery. In TIG-3 cells, acetylated p53 has been reported to increase at approximately 4 h after H_2_O_2_ exposure, followed by increases in p21 and later SA-β-gal activity [17]. Based on these observations, a practical time course would include baseline, 0, 4, 12, 24, and 48 h, with additional 72 h and 7-day measurements when longer-term recovery is of interest. The exact sampling schedule should be adjusted to the kinetics of the selected cell model and stressor.

Several measurements should be performed in parallel. SIRT1 functional state can be evaluated using total SIRT1 abundance, catalytic activity, substrate-specific deacetylation, and subcellular localization. Acetylated p53, FOXO3, and PGC-1α can provide complementary measures of substrate engagement [18]. NAD^+^ metabolism can be assessed using total cellular NAD^+^ and the NAD^+^/NADH ratio. Where technically feasible, compartment-specific NAD^+^ measurements using genetically encoded fluorescent biosensors can distinguish nuclear, cytoplasmic, and mitochondrial NAD^+^ pools [19]. This is important because bulk NAD^+^ measurements may not accurately reflect the local NAD^+^ environment available to SIRT1.

Redox and mitochondrial measurements should include ROS/RNS, mitochondrial membrane potential, respiratory function, and selected markers of mitochondrial quality control. Stress-response pathways can be followed using HSF1–HSP70, eIF2α–ATF4, and p53–p21, together with inflammatory markers such as NF-κB, IL-6, and IL-8 [23,24]. Recovery should be evaluated from the trajectory of these measurements rather than from a single endpoint. One useful measure would be the time required for a parameter to return toward its prestress range. A second stress challenge after partial or complete recovery could further test whether the initial challenge alters the magnitude or kinetics of a subsequent response.

Such measurements provide an experimental route for testing the proposed concept of SIRT1 functional reserve. Cells with relatively preserved reserve would be expected to mount a measurable SIRT1-dependent response and subsequently recover toward their prestress state. Cells with more constrained reserve may show prolonged stress signaling, incomplete recovery, or altered responses to a second challenge. These patterns would provide evidence for differences in adaptive capacity without assuming that reduced SIRT1 abundance alone represents loss of reserve. The interpretation should remain dependent on cell type, stressor, stress history, and the specific functional endpoint being examined.

### 7.3. Translational Challenges and Future Therapeutic Strategies

The main translational question is whether changes in SIRT1-dependent adaptive capacity can be linked to meaningful functional outcomes in humans. Clinical studies of SIRT1 activators and NAD^+^ precursors show that these pathways can be pharmacologically modified, but changes in biochemical or metabolic markers have not consistently produced broad functional improvements. This discrepancy makes it important to distinguish target engagement from restoration of cellular function.

The candidate biomarkers discussed above require prospective validation before they can be used for patient stratification. Several methodological problems remain. SIRT1 catalytic activity is not yet routinely measured in clinical samples, NAD^+^ metabolism differs among tissues and cellular compartments, and senescence-associated markers vary substantially among cell types and disease states. Future trials should therefore incorporate predefined biomarker analyses together with functional endpoints rather than relying solely on circulating NAD^+^ or a single molecular marker.

A more informative biomarker strategy may combine measurements of NAD^+^ metabolism, SIRT1-dependent deacetylation, mitochondrial function, and redox state [222,223]. Such a panel could potentially distinguish different biological constraints. For example, reduced NAD^+^ availability with relatively preserved downstream SIRT1 signaling would represent a different state from persistent mitochondrial dysfunction accompanied by impaired recovery. Whether these profiles predict treatment response remains unknown and requires prospective testing.

Therapeutic strategies should likewise account for the cellular state in which SIRT1 operates. Increasing SIRT1 activity may have different consequences depending on NAD^+^ availability, mitochondrial function, inflammatory signaling, protein turnover, and the degree of persistent cellular dysfunction. The relevant therapeutic question is therefore not simply whether SIRT1 activity can be increased, but whether the conditions required for an appropriate SIRT1-dependent response are present.

This distinction supports a more targeted approach to SIRT1 modulation. If NAD^+^ availability is limiting, NAD^+^-directed interventions may be relevant; if mitochondrial or redox dysfunction predominates, correcting these abnormalities may be necessary; and if persistent senescence-associated dysfunction has become established, SIRT1 modulation alone may be insufficient. These possibilities remain hypotheses rather than established treatment algorithms. Future clinical studies should examine tissue-level and functional outcomes together with biochemical target engagement and should determine whether specific biological profiles are associated with differential responses to intervention.

### 7.4. Concluding Perspective

The central proposition of this review is that SIRT1–redox biology is better understood as a dynamic component of cellular adaptation than as a linear antioxidant pathway. SIRT1 function is shaped by NAD^+^ metabolism, redox conditions, mitochondrial state, post-translational regulation, intracellular localization, substrate accessibility, and stress history. Aging can alter several of these factors simultaneously, so the same change in SIRT1 abundance or activity may have different consequences in different cellular environments.

SIRT1 functional reserve and adaptive capacity are proposed here as conceptual tools rather than established biological measurements. Their usefulness will depend on whether they can be translated into reproducible measurements that distinguish effective adaptation, reversible compensation, and persistent dysfunction. The stress-and-recovery experiments outlined above provide one possible way to test these concepts by examining SIRT1-dependent responses together with the cellular conditions that support or constrain them.

This perspective also changes how SIRT1-directed interventions might be evaluated. Rather than treating increased SIRT1 activity as an endpoint in itself, future studies should ask whether SIRT1-dependent responses are appropriate for the cellular state and whether they are followed by functional recovery. NAD^+^ metabolism, mitochondrial and redox function, proteostasis, inflammatory signaling, and persistent senescence-associated states may each constrain this process.

Advances in single-cell, spatial, and subcellular approaches should make it increasingly possible to examine these determinants at the level of individual cell populations and intracellular compartments. Such approaches may help determine when SIRT1-dependent adaptive capacity is constrained and whether the relevant constraint can be modified. Ultimately, the role of SIRT1 in aging is unlikely to be adequately described as simply protective or insufficient. Its effects depend on which cell is being examined, where SIRT1 is acting, what stress the cell has experienced, and whether recovery remains possible. Testing these relationships experimentally will be important for determining when modulation of SIRT1 can contribute to restoration of cellular adaptation and when additional components of the aging cellular environment must also be addressed.

## Figures and Tables

**Table 1:** Summary of the proposed experimental parameters, methods, and time points for operationalizing SIRT1 functional reserve.

Experimental Dimension	Measured Parameters	Methods	Time Points	References
SIRT1 functional state	Abundance, activity, substrate acetylation, localization	Western blot, fluorogenic assay, fractionation	Baseline, 0, 4, 12, 24, 48 h	[17,18]
NAD^+^ availability	Total NAD^+^, NAD^+^/NADH, compartment-specific NAD^+^	Cycling assay, LC–MS, biosensors	Same as above	[17,19]
Redox & mitochondria	ROS/RNS, membrane potential, respiration, mitophagy	MitoSOX, roGFP, Seahorse, immunoblot	Same as above	[20,21]
Mitochondrial complex I activity	Complex I oxygen consumption rate	Oroboros high-resolution respirometry	Same as above	[22]
Stress & recovery markers	HSF1–HSP70, eIF2α–ATF4, p53–p21, NF-κB	Western blot, qPCR, immunofluorescence	Same as above	[23,24]
Recovery kinetics	Time to baseline, reversibility	Longitudinal measurement	Throughout	[17,25]

**Table 3:** Comparative summary of SIRT1 responses across species, tissues, and stress paradigms.

Species	Tissue/Cell Type	Stress Paradigm	SIRT1 Response	Major Phenotype	Conservation	Representative References
Human	Fibroblasts (TIG-3)	H_2_O_2_	Reduced activity; NAD^+^ depletion	Senescence, p53 acetylation, p21 induction	Conserved	[17]
Human	Endothelial cells	Oxidative stress	Reduced expression; increased senescence	Endothelial dysfunction	Conserved	[116]
Human	Neural stem cells	Amyloid-β	Reduced SIRT1, PGC-1α, FOXO3	Senescence-like changes	Conserved	[130]
Mouse	Skeletal muscle	Caloric restriction/exercise	Tissue-specific changes in expression and activity	Metabolic remodeling	Tissue-specific	[90]
Mouse	Heart	Moderate vs. high SIRT1 overexpression	Dose-dependent effects	Protection at moderate levels; cardiomyopathy at high levels	Model-specific	[74]
Mouse	Liver	Aging and liver injury	Reduced hepatic SIRT1	NLRP3 activation, fibrosis	Conserved	[131]
Mouse	Adipose-derived stem cells	Aging	Reduced SIRT1; increased mitochondrial ROS	Impaired adipogenic capacity	Tissue-specific	[132]
Mouse	Microglia	Aging and neurodegeneration	Reduced SIRT1	IL-1β-associated inflammation, cognitive decline	Conserved	[133]
Zebrafish	Skeletal muscle	Glucocorticoid-induced atrophy	*Sirt1* inhibition impairs UPR^mt^	Protein aggregation, muscle deterioration	Model-specific	[68]
*C. elegans*	Whole organism	Hsp90 disruption	SIRT1 ortholog depletion	Proteasomal degradation	Conserved	[92]

**Table 4:** Selected clinical studies relevant to therapeutic modulation of the SIRT1–redox adaptive landscape.

Intervention	Population and Study Design	Regimen and Duration	Main Findings and Interpretive Limitations	References
SRT2104	Adults aged 60–80 years; randomized, double-blind, placebo-controlled phase I trial	0.5 or 2.0 g/day for 28 days	Short-term tolerability and favorable lipid changes were reported, without improved glucose tolerance. The short study duration does not establish sustained functional or anti-aging efficacy.	[203]
SRT2104	Adults with type 2 diabetes; randomized, double-blind, placebo-controlled crossover trial	2.0 g/day for 28 days per treatment period	Body weight decreased, but HbA1c increased by approximately 0.48 percentage points. Endothelial and fibrinolytic effects were inconsistent and predominantly neutral. The findings show potentially unfavorable metabolic effects in this population.	[204]
Nicotinamide riboside	Older adults with MCI; randomized, placebo-controlled pilot trial	Dose escalation to 1.0 g/day during a 10-week study	Blood NAD^+^ increased approximately 2.6-fold, without improvement in cognitive measures. Increased circulating NAD^+^ did not establish cognitive benefit or restoration of SIRT1-dependent adaptive capacity.	[206]
β-Nicotinamide mononucleotide	Older adults; randomized, double-blind, placebo-controlled trial	250 mg/day for 12 weeks	Blood NAD^+^ increased, but the primary stepping-test outcome was unchanged. Selected walking and sleep outcomes favored supplementation; these findings do not establish a general improvement in physical function.	[207]
Nicotinamide riboside with walking exercise	Sedentary middle-aged and older adults with hypertension; three-arm randomized pilot trial	NR 1.0 g/day with walking, placebo with walking, or NR alone for six weeks; walking for 30 min three times weekly	Adding NR to walking did not improve the primary daytime systolic blood pressure outcome compared with walking plus placebo. Biochemical responses did not establish additional antihypertensive efficacy.	[209]
MitoQ	Adults aged 65–80 years; randomized, double-blind, placebo-controlled trial	20 mg/day for 12 weeks	Maximal mitochondrial H_2_O_2_ emission capacity in skeletal muscle decreased, whereas mitochondrial respiration and several acute exercise redox responses were unchanged. Reduced oxidant emission did not establish improved adaptive recovery.	[211]
Dasatinib plus quercetin	Postmenopausal women; phase II randomized controlled trial	Intermittent treatment; primary assessment at 20 weeks	The primary CTx outcome did not differ significantly between groups. Early changes in bone-formation markers and signals in participants with higher senescence burden require cautious interpretation and further confirmation.	[212]
Dasatinib plus quercetin	Older adults with MCI and slow gait at risk for Alzheimer’s disease; single-arm pilot study	Dasatinib 100 mg plus quercetin 1250 mg on two days every two weeks for 12 weeks	The pilot supported treatment feasibility. Cognitive, mobility and biomarker observations were exploratory; the absence of a control group prevents conclusions about efficacy.	[213]

Abbreviations: CTx, C-terminal telopeptide of type I collagen; HbA1c, glycated hemoglobin; MCI, mild cognitive impairment; NAD^+^, oxidized nicotinamide adenine dinucleotide; NR, nicotinamide riboside; SIRT1, sirtuin 1. Note: None of the studies listed in this table stratified participants by SIRT1 functional reserve, tissue-specific NAD^+^ availability, or senescence-associated biomarkers. The largely negative or mixed outcomes should therefore be interpreted with the caveat that patient selection was not guided by the adaptive-capacity framework proposed in this review.

## Data Availability

No new data were created or analyzed in this study. Data sharing is not applicable to this article.
